# Efficacy and Safety of L‐Carnosine‐Containing Hyaluronate Injection for Facial Skin Hydration and Brightness: A Randomized, Controlled, Evaluator‐Blinded Trial

**DOI:** 10.1111/jocd.71030

**Published:** 2026-07-01

**Authors:** Zhanhong Li, Jingfang Sun, Zhehu Jin, Wei Guo, Aili Cui, Huaigu Wang, Jiahua Wang, Xiaoyi Qiu, Heyang Xi, Yao Zhang, Lu Zhang, Ligen Xuan, Lingzhi Shi, Yangliang Yi, Huiyin Qiu, Fangke Guan, Yaling Xie, Zhouwei Liu, Lili Liang, Haijun Yang, Chunfang Chen, Huiying Wang, Xiaoling Sun, Fengsheng Che, Qiong Meng

**Affiliations:** ^1^ Sihuan Pharmaceutical Holdings Group Ltd. Beijing China; ^2^ Beijing MEIYANKONGJIAN Biotechnology Co., Ltd. Beijing China; ^3^ Department of Medical Cosmetology Yanbian University Hospital Jilin China; ^4^ Department of Plastic Surgery Shulan (Quzhou) Hospital Quzhou China; ^5^ Department of Plastic Surgery and Burn The First Affiliated Hospital of Bengbu Medical University Bengbu China; ^6^ Department of Medical Cosmetology Ningbo Medical Center Lihuili Hospital Ningbo China; ^7^ Department of Cosmetic Dermatology Guangzhou Shuguang Medical Cosmetic Hospital Guangzhou China; ^8^ Department of Cosmetic Dermatology Liaoning Xinglin Plastic Surgery Hospital Shenyang China; ^9^ Department of Cosmetic Dermatology Guangzhou Huamei Medical Cosmetic Hospital Guangzhou China; ^10^ Department of Cosmetic Dermatology Foshan Shuguang Jinzi Medical Cosmetic Hospital Foshan China; ^11^ Department of Cosmetic Dermatology Liaoning Shuguang Plastic Surgery Hospital Shenyang China; ^12^ Department of Cosmetic Dermatology Shenzhen Fuhua Medical Cosmetic Hospital Shenzhen China; ^13^ Department of Cosmetic Dermatology Changchun Zhongyan Oracle Medical Cosmetic Hospital Changchun China; ^14^ Department of Cosmetic Dermatology Heilongjiang Chaolong Medical Cosmetic Hospital Harbin China; ^15^ Department of Cosmetic Dermatology Wuhan Liancheng Yimeiyangyan Medical Cosmetic Clinic Wuhan China; ^16^ Department of Cosmetic Dermatology Shenzhen Boerman Medical Cosmetic Clinic Shenzhen China; ^17^ Department of Cosmetic Dermatology Foshan Huamei Plastic Surgery Hospital Foshan China; ^18^ Department of Cosmetic Dermatology Shenzhen MARPHIL Medical Cosmetic Clinic Shenzhen China

**Keywords:** hyaluronic acid, L‐carnosine, mesotherapy, skin quality, superiority clinical trial

## Abstract

**Background:**

L‐carnosine, a dipeptide with superior antioxidant and antiglycation properties, serves as the core active component in this study. We aimed to evaluate the efficacy and safety of a novel injectable composite solution, which features L‐carnosine in combination with non‐crosslinked sodium hyaluronate, glycine, alanine, proline, and vitamin B_2_, for improving facial skin dryness and dullness.

**Methods:**

In this prospective, multicenter, randomized, evaluator‐blinded, superiority clinical trial, 466 subjects were randomized 1:1 to receive either the composite solution injections every 4 weeks for three sessions (treatment group) or no intervention (control group). The control group served as a baseline reference for natural temporal changes. Efficacy was assessed using the Global Aesthetic Improvement Scale (GAIS), the Skin Dryness and Dullness Improvement Scale (SDDIS), the Allergan Fine Lines Scale (AFLS), and the Allergan Skin Roughness Scale (ASRS). A predefined superiority margin of 15% was set for between‐group differences. Blinded evaluators used standardized photographs to score the scales. Safety was evaluated based on adverse events.

**Results:**

From baseline to Week 12, the treatment group exhibited significantly superior improvements in GAIS, SDDIS, AFLS, and ASRS scores relative to the control group. Efficacy assessed at Week 12 further highlighted this advantage (GAIS: 86.49% vs. 5.26%, SDDIS: 82.88% vs. 5.70%, AFLS: 36.07% vs. 4.57%, ASRS: 47.49% vs. 5.02%; all *p* < 0.05). These findings were consistent across both full analyses and per‐protocol analyses. Participant satisfaction was significantly higher in the treatment group (*p* < 0.05). Treatment‐related adverse events occurred in 94/228 (41.23%) of treated participants, predominantly mild injection‐site reactions (e.g., erythema, swelling) that resolved within 2 weeks.

**Conclusion:**

This randomized trial demonstrated that the LIVIGI Sodium Hyaluronate Composite Solution for Injection provided favorable clinical efficacy and acceptable safety in improving facial skin dryness and dullness, suggesting its potential as a promising option for skin quality enhancement in aesthetic practice. Furthermore, these findings underscore the growing role of multi‐component synergistic formulations in the evolving field of mesotherapy.

## 1 Introduction

Facial aesthetic appearance, particularly skin quality, exerts a profound impact on an individual's psychological well‐being and social confidence. Skin aging represents a multifaceted biological process, driven not only by intrinsic factors such as endogenous genetics, hormonal fluctuations, and the natural decline in cellular function, but also markedly accelerated by extrinsic exposures including ultraviolet radiation (UV), environmental pollutants, and unhealthy lifestyle choices [1, 2]. Together, these internal and external factors reduce synthesis and enhance degradation of skin extracellular matrix (ECM) components, leading to progressive structural deterioration and functional impairment [3].

Among ECM components, HA is essential for maintaining dermal hydration and biomechanical stability owing to its strong water‐retention capacity [4, 5]. Declining HA levels during aging contribute to increased transepidermal water loss and impaired skin barrier function [6, 7]. In parallel, oxidative stress and chronic low‐grade inflammation accelerate matrix degradation and disrupt pigment metabolism through the upregulation of matrix metalloproteinases and melanogenesis‐related pathways [8, 9, 10]. Consequently, therapeutic interventions aimed at restoring the ECM architecture, particularly those that enhance its hydration status and support microenvironmental metabolic homeostasis, are feasible pathways for improving aging phenotypes such as dryness and dullness [11].

Mesotherapy using non‐crosslinked HA has been widely adopted for skin quality improvement because of its favorable biocompatibility, biodegradability, and diffusion properties within the dermal ECM [12, 13]. Unlike crosslinked HA fillers primarily intended for volumization, non‐crosslinked HA formulations are mainly used to improve hydration, texture, and skin radiance [14, 15]. However, conventional HA monotherapy primarily addresses hydration and may have limited capacity to simultaneously target oxidative stress, collagen degradation, and the accumulation of advanced glycation end products (AGEs) associated with skin aging [16, 17]. L‐carnosine, an endogenous dipeptide with potent antioxidative and anti‐glycation properties, has been shown to protect collagen from oxidative and glycation‐induced damage through scavenging reactive oxygen species (ROS) and inhibiting the formation of AGEs [18]. Previous studies have further demonstrated that the combination of L‐carnosine with HA may enhance protection against UVB‐induced photoaging [19]. In addition, amino acids serve as essential substrates for collagen biosynthesis and dermal matrix remodeling. Shi A et al. reported that supplementation with glycine, alanine, and proline significantly enhanced the moisturizing efficacy of HA‐based formulations [20]. Collectively, these findings suggest that incorporating functional bioactive components into HA‐based formulations may provide synergistic benefits across multiple dimensions of skin rejuvenation [21, 22].

LIVIGI Sodium Hyaluronate Composite Solution for Injection (National Medical Products Administration Approval No. 20253132016, Sihuan Pharmaceutical Holdings Group Ltd., China) is a novel non‐crosslinked HA formulation that incorporates L‐carnosine, glycine, alanine, proline, and vitamin B2, stabilized within a phosphate‐buffered system (pH 6.8–7.4). Compared with conventional HA‐based mesotherapy products, this formulation was designed to enhance hydration, antioxidative defense, anti‐glycation activity, and collagen synthesis support, thereby more comprehensively addressing clinical demands for improvements in skin tone, texture, and radiance. Therefore, we conducted a prospective, multicenter, randomized, controlled, assessor‐blinded superiority trial to evaluate the efficacy and safety of this novel formulation in improving facial skin dryness and dullness.

## 2 Methods

### 2.1 Subjects

**FIGURE 1:**
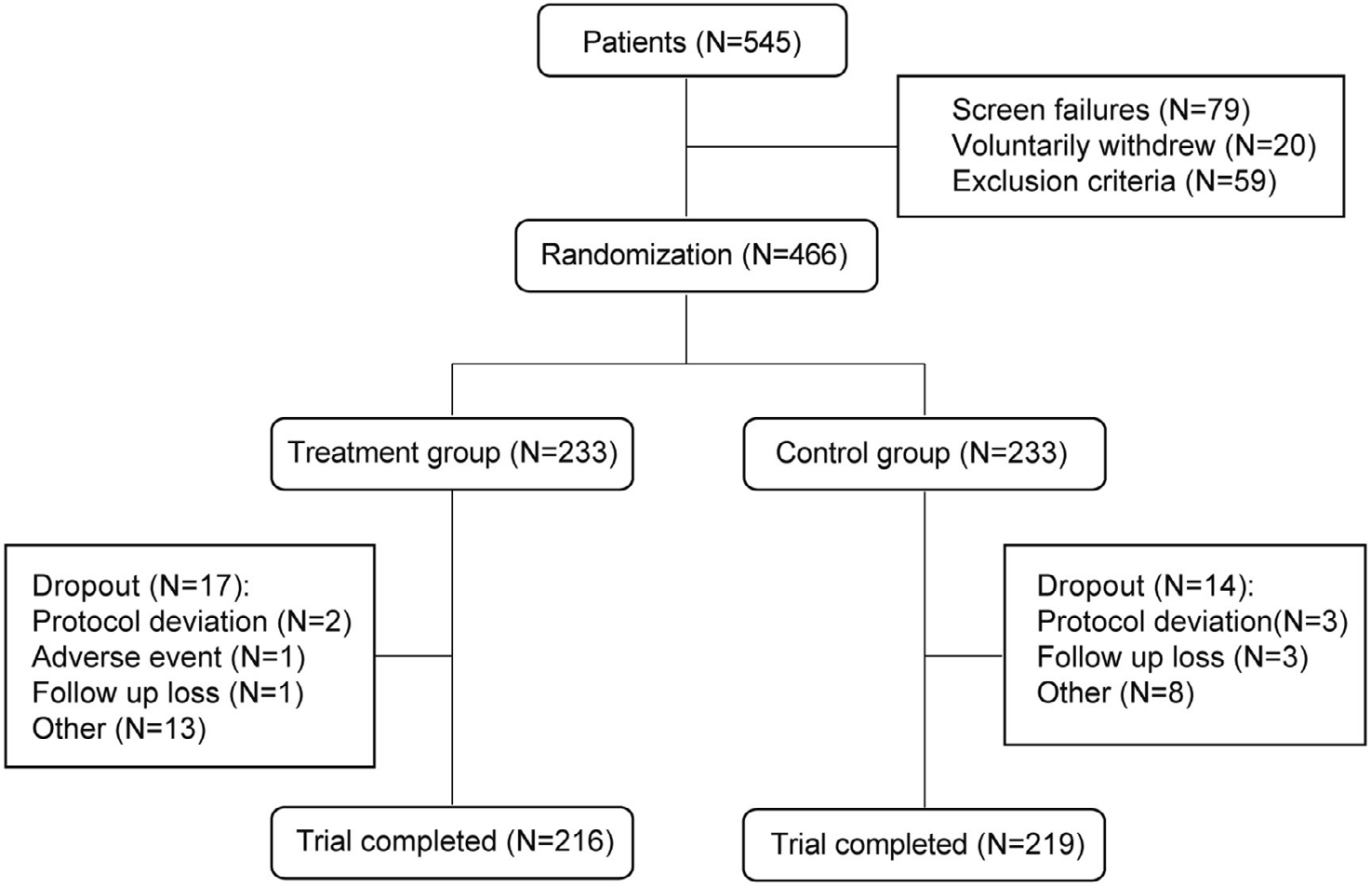
Flowchart of subjects distribution in the trial.

The trial was conducted at five clinical centers in China: Yanbian University Hospital, Shulan (Quzhou) Hospital, People's Hospital of Quzhou City, First Affiliated Hospital of Bengbu Medical University, and Ningbo Medical Center Lihuili Hospital. From February 20, 2024, to November 1, 2024, we enrolled 466 healthy subjects aged 18 to 65 years with Fitzpatrick skin phototype II‐IV, who were seeking improvement in facial skin dryness and/or dullness. All participants provided written informed consent and committed to adhering to the study protocol for the complete treatment and follow‐up schedule (Figure 1).

Exclusion criteria comprised individuals with a history of abnormal wound healing or keloid formation; those with active facial skin inflammation, infection, or non‐healed wounds; individuals known hypersensitivity to any component of the investigational product or local anesthetics; recipients of recent facial aesthetic procedures (e.g., filler injections, energy‐based devices, or cosmetic surgery) or use of immunosuppressive/anticoagulant therapy; patients diagnosed with severe systemic conditions such as malignancies, autoimmune diseases, significant organ dysfunction, or coagulation disorders and individuals who were pregnant, lactating, or planning a pregnancy during the study period.

This study adopted a block randomization design using an IWRS‐based dynamic randomization system, ensuring that treatment allocation was unpredictable to both investigators and subjects. Eligible subjects were randomized in a 1:1 ratio to a no‐treatment control group or a treatment group administered the LIVIGI sodium hyaluronate composite solution. Participants in the treatment group received a total of 3 injections, with 4‐week intervals between sessions. Follow‐up assessments for this group were conducted at 1 month and 3 months after the final injection. Subjects in the control group were evaluated at Weeks 4, 8, and 12.

### 2.2 Treatment

Control group subjects received no intervention. Subjects in the treatment group received three sessions of intradermal injections of LIVIGI sodium hyaluronate composite solution, spaced 4 weeks apart (the manufacturer's recommended protocol for full‐face mesotherapy). The injection protocol was standardized across all centers. All injecting physicians underwent standardized on‐site training before the trial, and a procedural checklist was used at each visit. Before each session, topical anesthetic cream was applied for 30–40 min, followed by disinfection. Using a Derma Shine electronic injector with a 32G needle (Specification: Panace‐DS‐30, National Medical Products Administration Approval No. 20192141528, Beijing Jingzhi Pharmaceutical Technology Co. Ltd., China), the product was injected into the facial dermis (depth 0.6–1.2 mm) at 0.02–0.05 mL per point, with a 5 mm spacing, to a total volume of 4 mL per session. The injection sequence (chin, cheeks, forehead) was fixed. A medical cold compress was applied post‐treatment immediately for 10–20 min. Subjects were instructed to avoid wearing makeup for 12 h and to avoid prolonged exposure to sunlight, ultraviolet rays, frost, as well as saunas or hot baths for two weeks following the injection.

### 2.3 Efficacy Evaluation

During the study period, standardized photographs were periodically submitted to three independent blinded evaluators for assessment of the Global Aesthetic Improvement Scale (GAIS) and the Skin Dryness and Dullness Improvement Scale (SDDIS). The SDDIS was developed based on the evaluation framework of the GAIS (Table 1). Detailed information regarding the development of the SDDIS and its assessment principles is provided in the Supporting Information. Blinded evaluators were not involved in trial procedures and had no access to information or documents that could potentially compromise blinding throughout the study. The primary endpoint was defined as whether the efficacy rates of GAIS/SDDIS scores at Week 12 after randomization were superior in the treatment group compared with the control group. The response rate was defined as the proportion of subjects rated as “very much improved,” “much improved,” or “improved” among all subjects who underwent GAIS/SDDIS evaluation. The secondary endpoints included the efficacy rates of the GAIS scores and SDDIS scores, which were assessed by investigators at Week 4, 8, and 20, as well as the Allergan Fine Lines Scale (AFLS) scores and the Allergan Skin Roughness Scale (ASRS) scores, which were assessed at Week 4, 8, 12, and 20 (Tables 2 and 3) [23, 24]. Additionally, a five‐point scale was used to assess patients' satisfaction with the final treatment outcome.

**TABLE 1:** Global aesthetic improvement scale (GAIS)/Skin dryness and dullness improvement scale (SDDIS).

Grade	Notes
3	Very much improved
2	Much improved
1	Improved
0	No change
−1	Worse

**TABLE 2:** Allergan fine lines scale (AFLS).

Grade	Notes
0 (Note)	No fine lines
1 (Minimal)	1–2 superficial lines
2 (Moderate)	3–5 superficial lines
3 (Severe)	Greater than 5 superficial lines; no crosshatching
4 (Diffuse)	Diffuse superficial lines; crosshatching

**TABLE 3:** Allergan skin roughness scale (ASRS).

Grade	Notes
0 (Note)	Smooth visual skin texture
1 (Minimal)	Slightly coarse and uneven visual skin texture
2 (Moderate)	Moderately coarse and uneven visual skin texture; may have early elastosis
3 (Severe)	Severely coarse visual skin texture, crosshatched fine lines; may have some elastosis
4 (Extreme)	Extremely coarse visual skin texture, crosshatched deep creases; extreme elastosis

### 2.4 Safety Evaluation

Baseline demographic data (including gender and age) were collected for subjects in both groups. Vital signs, including blood pressure, heart rate, and body temperature, were monitored before and after injection. Adverse events (AEs) and serious adverse events (SAEs) were documented, summarized by patient count, incidence, and percentage.

### 2.5 Sample Size Determination

Based on expert clinical judgment, the response rate at 1 month after the final injection was estimated to be 65% in the treatment group, whereas the corresponding response rate in the control group at 1 month after randomization was estimated to be approximately 25%. A superiority margin of 15% was predefined, with a statistical power of 1 − *β* = 0.90 and a one‐sided significance level of *α* = 0.025. The allocation ratio between the treatment and control groups was set at 1:1. Using PASS 11.0 software, a required sample size of 72 subjects per group was calculated. After accounting for an anticipated dropout rate of 20%, the adjusted sample size was increased to 90 subjects per group.

Furthermore, according to the Technical Review Guidelines for Clinical Trials of Sodium Hyaluronate Composite Solutions (Draft for Comments), at least 400 subjects (200 subjects each in the treatment and control groups) are required to adequately evaluate product safety and efficacy. Therefore, the final sample size was set at 460 subjects, with 230 subjects allocated to each group.

### 2.6 Statistical Analysis

This study defined three analysis populations: (1) The full analysis set (FAS) included all enrolled subjects with at least one post‐baseline GAIS and SDDIS assessment, with exclusions made only in special circumstances. For the experimental group, this was further restricted to those who used the investigational medical device at least once. (2) The per‐protocol set (PPS) was a subset of the FAS, comprising subjects who adhered to the prescribed treatment protocol and had primary endpoint data available. (3) The Safety Set (SS) included all enrolled subjects who underwent at least one safety evaluation. In the experimental group, subjects were required to have used the investigational medical device at least once.

Efficacy analyses were primarily based on the FAS. Missing primary endpoint data were imputed using the Worst Observation Carried Forward (WOCF) method. The PPS analysis was conducted without missing data imputation and was used to assess the consistency and robustness of the efficacy results obtained from the FAS analysis. No multiplicity adjustment was applied to secondary endpoints, and their results are interpreted as exploratory. Statistical analysis was performed using SAS 9.4 software. All statistical tests were two‐sided, and *p* < 0.05 was considered statistically significant. Parameter estimates are presented with two‐sided 95% confidence intervals (CI). Quantitative variables were summarized using descriptive statistics, including the number of observations, missing values, mean, standard deviation, median, first quartile, third quartile, minimum, and maximum. Categorical variables were described as numbers and percentages. Between‐group comparisons were performed using the *T*‐test or Wilcoxon rank‐sum test for quantitative variables, the Chi‐square test or Fisher's exact test for categorical variables (if the chi‐square test is not applicable), and the Wilcoxon rank‐sum test or Cochran–Mantel–Haenszel (CMH) test for ordinal data.

## 3 Results

### 3.1 Subjects

A total of 466 subjects were enrolled in this trial. Among them, 450 subjects were included in the FAS, with 222 subjects in the treatment group and 228 subjects in the control group. During the study period, 31 subjects discontinued prematurely for various reasons. The PPS consisted of 411 subjects, including 203 subjects in the treatment group and 208 subjects in the control group. The SS included 461 subjects, with 228 subjects in the treatment group and 233 subjects in the control group. The sample sizes for each set met the statistical requirements specified in the protocol.

Demographic and baseline characteristics were similar between the treatment group and the control group, as summarized in Table 4. Based on FAS analysis, the mean age was 34.78 ± 8.78 years in the treatment group and 35.05 ± 9.30 years in the control group (*p* > 0.05). In the treatment group, 17 subjects (7.66%) were male and 205 (92.34%) were female; in the control group, 17 subjects (7.46%) were male and 211 (92.54%) were female (*p* > 0.05). The mean height was 162.17 ± 6.01 cm in the treatment group and 162.02 ± 6.04 cm in the control group (*p* > 0.05). Similarly, the mean weight was comparable between groups, measuring 58.46 ± 9.62 kg in the treatment group and 58.20 ± 9.30 kg in the control group (*p* > 0.05).

**TABLE 4:** Demographic data.

Parameter	Indicator	Experimental group	Control group	Total	Statistic	*p*
Age (years)	*N* (Missing)	222 (0)	228 (0)	450 (0)	*T* test −0.315	0.753
	Mean (SD)	34.78 (8.78)	35.05 (9.30)	34.92 (9.04)		
	Median	33.5	34	34		
	Q1, Q3	28, 40	28, 40	28, 40		
	Min, Max	19, 62	20, 63	19, 63		
Gender	Female, *n* (%)	205 (92.34%)	211 (92.54%)	416 (92.44%)	Chi‐square test 0.007	0.936
	Male, *n* (%)	17 (7.66%)	17 (7.46%)	34 (7.56%)		
Height (cm)	*N* (Missing)	222 (0)	228 (0)	450 (0)	*T* test 0.274	0.784
	Mean (SD)	162.17 (6.01)	162.02 (6.04)	162.09 (6.02)		
	Median	161.55	161.05	161.5		
	Q1, Q3	158, 165	158, 165	158, 165		
	Min, Max	147.5, 180	147.5, 184	147.5, 184		
Weight (kg)	*N* (Missing)	222 (0)	228 (0)	450 (0)	*T* test 0.298	0.766
	Mean (SD)	58.46 (9.62)	58.20 (9.30)	58.33 (9.45)		
	Median	56.75	57	57		
	Q1, Q3	52, 64	51, 64	52, 64		
	Min, Max	38, 96	36, 104.5	36, 104.5		

Based on the FAS analysis, a history of medical conditions was reported in 28 subjects (12.61%) in the treatment group and 34 subjects (14.91%) in the control group, as shown in Table 5. No statistically significant difference was observed between the two groups (*p* > 0.05). With respect to allergy history, 3 subjects (1.35%) in the treatment group and 4 subjects (1.75%) in the control group reported prior allergic conditions, with no significant between‐group difference observed (*p* > 0.05).

**TABLE 5:** Past medical history.

Parameter	Indicator	Experimental group	Control group	Total	Statistic	*p*
Medical history	Present, *n* (%)	28 (12.61%)	34 (14.91%)	62 (13.78%)	Chi‐square test	0.479
	None, *n* (%)	194 (87.39%)	194 (85.09%)	388 (86.22%)	—	
Allergy history	Present, *n* (%)	3 (1.35%)	4 (1.75%)	7 (1.56%)	Fisher's exact test	1.000
	None, *n* (%)	219 (98.65%)	224 (98.25%)	443 (98.44%)	—	

### 3.2 Primary Efficacy Endpoint

**FIGURE 2:**
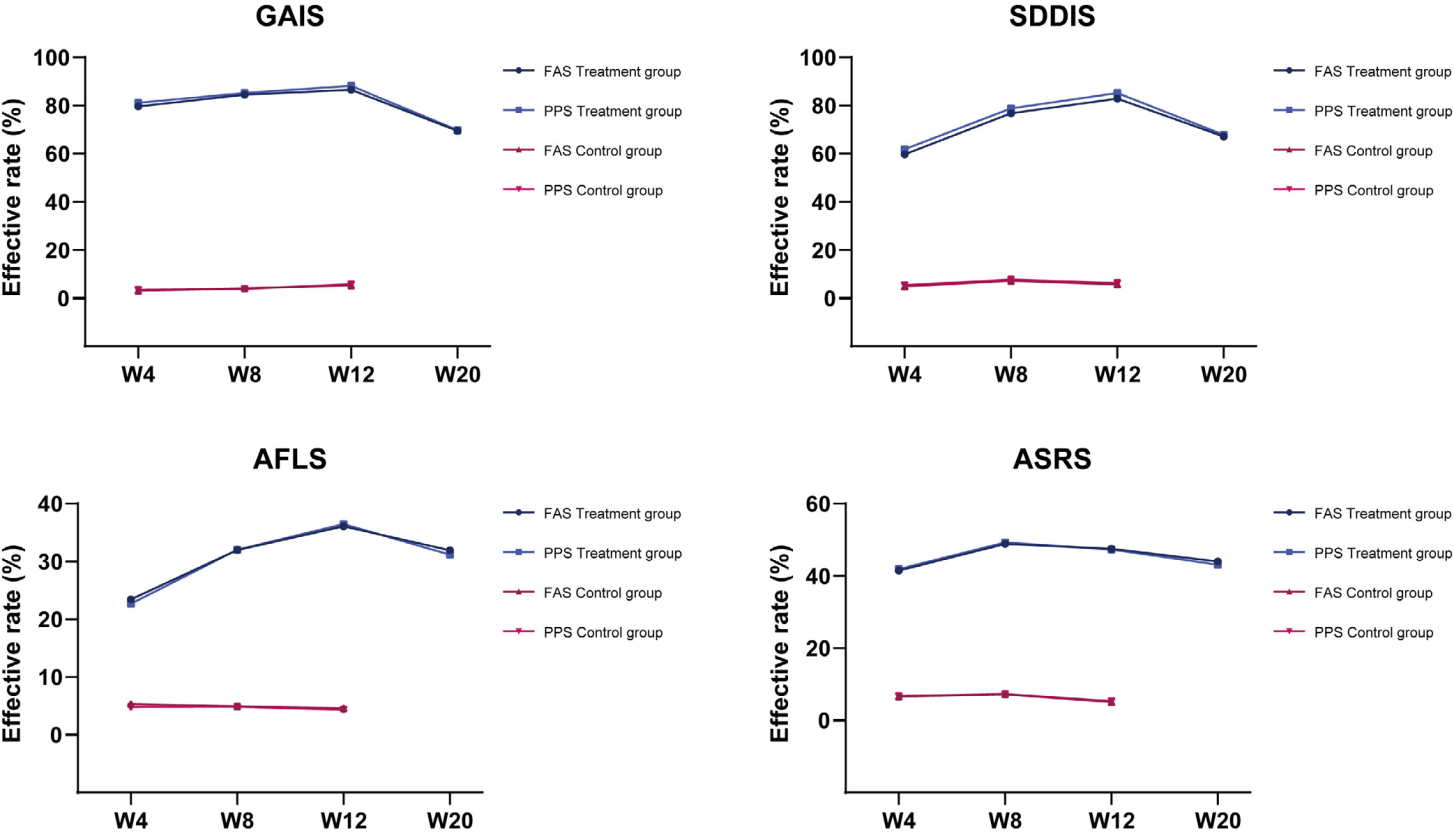
Efficacy rates of GAIS/SDDIS scores (independently assessed) and AFLS/ASRS scores (investigator assessment) in the treatment group and the control group, including both FAS and PPS populations. W4, week 4; W8, week 8; W12, week 12; W20, week 20.

**FIGURE 3:**
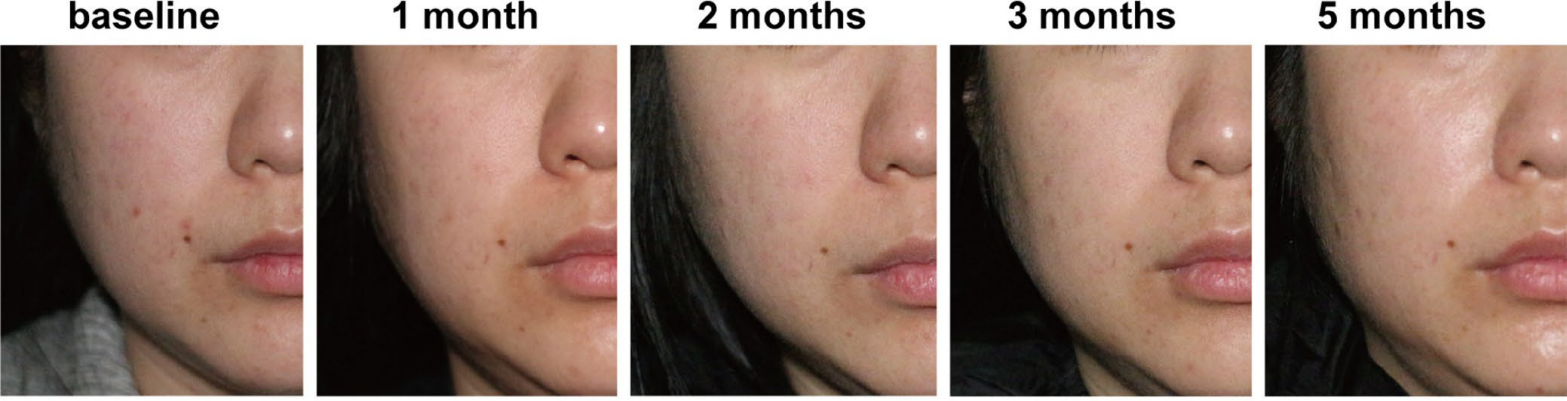
Clinical imaging records of representative case in the treatment group.

Based on FAS and PPS analyses, the treatment group demonstrated significantly higher efficacy rates than the control group for both GAIS and SDDIS scores at Weeks 12 and had a statistically significant difference (Figure 2). Based on FAS analysis, the efficacy rates for the GAIS scores and the SDDIS scores in the treatment group were 86.49% and 82.88%, respectively, compared to 5.26% and 5.70% in the control group. The 95% CI for the between‐group differences in efficacy was 81.2% (75.9%, 86.6%) and 77.2% (71.4%, 83.0%), respectively, both exceeding the pre‐specified superiority margin of 15% (Table 6). The PPS results were consistent with those of the FAS, indicating that the treatment group demonstrated superior efficacy compared to the control group. Figure 3 illustrates a representative case from the treatment group. The subject exhibited noticeable improvement in skin condition after the first treatment session, with the most pronounced enhancement observed after three treatment sessions. Pre‐existing facial dryness, erythema, and dullness were markedly alleviated, and the overall skin appearance became more luminous, better hydrated, and more even in texture.

**TABLE 6:** Proportion of subjects meeting the improvement criteria for GAIS and SDDIS at Week 12 as assessed by third‐party evaluators.

GAI (full analysis set)	Treatment group (*N* = 222)	Control group (*N* = 228)	Statistic	*p*
Responders, *n* (%)	192 (86.49)	12 (5.26)	Chi‐square test	< 0.001
Between‐group difference, % (95% CI)	81.2% (75.9%, 86.6%)			
Superiority	Yes			

SDDIS (full analysis set)	Treatment group (*N* = 222)	Control group (*N* = 228)	Statistic	*p*
Responders, *n* (%)	184 (82.88)	13 (5.70)	Chi‐square test	< 0.001
Between‐group difference, % (95% CI)	77.2% (71.4%, 83.0%)			
Superiority	Yes			

### 3.3 Secondary Efficacy Endpoint

Based on FAS analysis, the GAIS efficacy rate in the treatment group was 79.64% and 84.55% at Weeks 4 and 8, respectively, compared to 3.10% and 4.04% in the control group. Similarly, the SDDIS efficacy rate in the treatment group reached 59.73% and 76.82% at Weeks 4 and 8, respectively, compared to 4.87% and 7.17% in the control group. Based on FAS analysis, the AFLS efficacy rate in the treatment group increased progressively from 23.42% at Week 4 to 31.96% at Week 8 and 36.07% at Week 12, whereas the corresponding rates in the control group remained low at 5.29%, 4.93%, and 4.57%, respectively. Consistent results were observed for the ASRS, with efficacy rates of 41.44%, 48.86%, and 47.49% in the treatment group at Weeks 4, Weeks 8, and Weeks 12, respectively, versus 6.61%, 7.17%, and 5.02% in the control group. Analyses based on the PPS yielded results comparable to those of the FAS for all four outcome measures, and statistically significant differences between the treatment and control groups were consistently observed at each time point (*p* < 0.001). At Week 12, 86.31% of subjects in the treatment group reported being “satisfied” or “very satisfied” with the treatment outcome, compared to 67.89% in the control group (*p* < 0.05). In summary, the treatment group demonstrated significant superiority over the control group in GAIS, SDDIS, AFLS, and ASRS scores as well as subject satisfaction. Additionally, a noteworthy observation was that the efficacy rates showed an upward trend during continuous treatment, followed by a decline after the treatment cycle (Week 20). Consistently, the proportion of subjects in the treatment group who reported being “satisfied” or “very satisfied” with treatment outcomes decreased from 86.31% at 4 weeks after the last injection (Week 12) to 78.24% at 3 months after the last injection (Week 20). This decline was potentially attributed to the gradual degradation of sodium hyaluronate within the body over time following the treatment cycle, which led to a progressive reduction in treatment efficacy. Nevertheless, considering the potential influence of participant expectations in this non‐blinded setting, satisfaction‐related findings should be interpreted with caution.

### 3.4 Safety

The severity of adverse events was graded on a 4‐point scale (Grades 0–3): 0, none or not applicable; 1, mild, with noticeable signs or symptoms that were easily tolerated; 2, moderate, causing sufficient discomfort to interfere with daily activities; 3, severe, resulting in incapacity and inability to work or perform normal daily activities. No device‐related adverse events were observed in the control group because no treatment was administered. 436 AEs (41.23%, 94/228) related to the investigational test device were reported in the treatment group (*N* = 228) and 90.74% (392/432) were classified as Grade 0–1, while 9.26% (40/432) were classified as Grade 2. All events were common injection site reactions, including erythema, pain, swelling, hemorrhage, and edema (Table 7). Most adverse events (97.22%, 420/432) resolved within 14 days. By the end of the study, 99.77% (431/432) of adverse events had completely resolved, with the remaining event showing improvement. No other injection‐related complications, such as infection, hypersensitivity reactions, or vascular occlusion, were observed. No serious device‐related AEs were reported nor were any clinically significant abnormalities in vital signs (blood pressure, pulse, or body temperature) observed throughout the trial.

**TABLE 7:** AEs (injection site reactions) in the treatment group.

AE	Number of AE	Number of patients	Percentage
Erythema	141	70	30.70
Pain	75	42	18.42
Swelling	73	42	18.42
Hematoma	77	37	16.23
Edema	39	28	12.28
Pruritus	12	10	4.39
Acne	6	5	2.19
Discoloration	3	3	1.32
Epidermal exfoliation	4	2	0.88
Rash	1	1	0.44
Bruising	1	1	0.44

## 4 Discussion

Skin dryness and dullness are common manifestations of facial skin aging and are closely associated with alterations in dermal ECM homeostasis [25, 26]. External stimuli such as UV can induce oxidative stress and inflammatory responses, impair fibroblast function, and upregulate matrix metalloproteinases (MMPs), thereby accelerating ECM degradation and compromising the structural integrity of the dermis [27]. Reduced skin hydration and impaired barrier function lead to increased transepidermal water loss (TEWL) [28]. In addition, abnormal activation of melanocytes disrupts melanin synthesis and transport, resulting in uneven skin tone and dullness [29, 30].

In recent years, as patient expectations have extended beyond simple hydration, injectable “skin boosters” based on HA and purposefully combined with multiple bioactive ingredients have attracted increasing attention for skin quality improvement [31]. Through ongoing formulation development, these products are gradually evolving into multifunctional therapeutic systems, offering broader benefits such as skin brightening and anti‐wrinkle effects [32, 33].

This study evaluated the effects of a novel injectable non‐crosslinked sodium hyaluronate composite solution containing L‐carnosine on improving facial skin dryness and dullness. The results demonstrated that improvements in facial skin dryness and dullness were significantly greater in the treatment group than in the untreated control group, and these improvements were consistently observed across investigator‐assessed efficacy rates and multiple subjective evaluation scales, including GAIS, SDDIS, AFLS, and ASRS. Notably, clinical improvement became apparent after the first treatment session and gradually increased with repeated injections, suggesting a cumulative treatment effect. Although efficacy showed a mild decline at 3 months after the final treatment, treatment responses remained higher than baseline levels, indicating a certain degree of persistence during the follow‐up period.

The observed clinical improvements may be related to the combined effects of the formulation components. HA is known to improve dermal hydration and may contribute to ECM support and skin viscoelasticity [34]. These effects may improve stratum corneum smoothness and light reflectance, thereby reducing skin roughness and enhancing skin radiance [35]. L‐carnosine, a key component of this formulation, exhibits antioxidant, antiglycation, and senolytic‐related properties. Previous studies have shown that it can alleviate UV‐induced photoaging by enhancing mitochondrial activity, reducing ROS production and AGEs accumulation [36, 37, 38]. Li X et al. have verified that L‐carnosine stimulated macrophage‐mediated clearance of senescent dermal fibroblasts and human keratinocytes through activation of the CD36/AGE receptor‐AKT2 signaling pathway [39]. Additionally, several studies have shown that topical formulations or sodium hyaluronate injectables containing carnosine may improve skin elasticity, pigmentation, and wrinkles, which is similar to the findings of the present study [40, 41]. Glycine, proline, and alanine are essential components for stabilization of the collagen triple‐helix structure and important constituents of the natural moisturizing factor [42, 43, 44, 45]. Adding them to the formula may contribute to collagen synthesis and tissue repair. The formulation also includes vitamin B2, an essential nutrient reported to participate in cellular redox reactions and maintain normal cellular energy metabolism [46]. Deficiency of vitamin B2 has been reported to be associated with skin inflammation and may contribute to the development of telangiectasia and erythema [47].

In conclusion, based on an innovative multi‐component synergistic design, this compound solution demonstrated favorable clinical effects on skin quality. Notably, 86.31% of treated participants reported a marked improvement in skin condition, particularly in facial dryness, erythema, and dullness, accompanied by subjective perceptions of enhanced skin radiance and hydration. Previous studies have primarily emphasized the potent hydrating and moisturizing properties of non‐crosslinked HA, which likely contributed substantially to the observed improvements in skin hydration and texture. In contrast, the improvements in facial erythema, dullness, and skin radiance may involve biological mechanisms beyond hydration alone and could be associated with the synergistic effects of L‐carnitine, accompanying amino acids, and vitamin B2. These components have been reported to exhibit antioxidant, anti‐glycation, metabolic‐supporting, and skin‐repair‐related properties, which may contribute to the enhancement of overall skin quality. Nevertheless, because an HA‐only comparator group was not included, the specific contribution of these additional components could not be definitively distinguished in the current study. Regarding safety, transient injection‐site reactions were relatively common, primarily presenting as erythema and swelling, which are associated with the use of electronic injection devices. These reactions were generally mild, well tolerated, and considered expected post‐procedural events, resolving within approximately two weeks with routine postoperative care. No serious AEs related to the study product were reported, and no product defects occurred.

We acknowledge several limitations of this study. First, the efficacy assessment relied primarily on subjective evaluations, lacking objective measurement indicators. However, subjective perception‐based scales are widely accepted in aesthetic medicine for the evaluation of clinical outcomes. In addition, the study employed a rigorous analytical approach, including standardized photographic documentation and blinded third‐party evaluations. Second, the follow‐up period was relatively short, and the study population consisted predominantly of female participants and subjects with Fitzpatrick skin types II‐IV. As this demographic profile is generally representative of the population seeking aesthetic treatments in clinical practice, the findings are most directly applicable to this group, while further studies in more diverse populations would be valuable to confirm their broader applicability. Third, a direct control group receiving a single HA‐based injectable formulation was not included, primarily because the current phase focused on validating the efficacy and safety of the combination formula. Future studies should incorporate objective parameters such as corneometry and skin roughness, extend the follow‐up duration, and include comparisons with single HA‐based injectable formulations or other mesotherapy products to further validate and extend these findings.

## 5 Conclusion

In conclusion, the LIVIGI Sodium Hyaluronate Composite Solution for Injection demonstrated favorable clinical efficacy and acceptable safety in improving facial skin dryness and dullness, suggesting that this formulation may represent a promising option for skin quality improvement in aesthetic practice. These findings also support the evolving concept of mesotherapy from single‐agent delivery toward synergistic, multi‐component formulations. Future studies should incorporate objective outcome measures, longer follow‐up periods, and direct comparisons with single‐agent HA formulations or other mesotherapy products to further clarify its relative advantages and broader applicability.

## Author Contributions

Conceptualization: F.C. and Q.M. Methodology: J.S., X.Q., H.X., Y.Z. and L.Z. Validation: Z.L., L.L., H.Y., C.C., H.W. and X.S. Formal analysis: L.X., L.S., Y.Y., H.Q., F.G. and Y.X. Investigation: Z.J., W.G., A.C., H.W. and J.W. Data curation: Z.L., J.S. and Z.J. Writing – original draft preparation: Z.L. and Q.M. Writing – review and editing: Z.L., F.C., Z.J. and Q.M. Visualization: Z.L. and J.S. Supervision: J.S., Z.J., F.C. and Q.M.

## Funding

This noninterventional study was funded by Sihuan Pharmaceutical Holdings Group Ltd. (Beijing, China), which provided financial support for administrative and documentation costs only.

## Ethics Statement

The clinical trial protocol was approved by the Yanbian University Hospital Ethics Committee (No. 2024007). The protocol was approved by the Shulan (Quzhou) Hospital Ethics Committee (No. 2024001). The protocol was approved by the Ethics Committee of the People's Hospital of Quzhou City (No. 2024005). The protocol was approved by the Ethics Committee of the First Affiliated Hospital of Bengbu Medical University (No. 2024‐043‐X01). The protocol was approved by the Ningbo Medical Center Lihuili Hospital Ethics Committee (No. QX2024YJ020‐03).

## Consent

For all personal information used in this article, for example, personal images, a consent for publication has been obtained.

## Conflicts of Interest

The authors declare no conflicts of interest.

## Supporting information


**Table S1:** Subject skin assessment scale.
**Table S2:** Improvement grading of transient skin dryness and facial dullness (third‐party evaluator vs. investigator).
**Table S3:** Response rate of improvement in transient skin dryness and facial dullness (third‐party evaluator vs. investigator).

## Data Availability

The data that support the findings of this study are available from the corresponding author upon reasonable request.
