# Supplementary material for: Efficacy and Safety of L‐Carnosine‐Containing Hyaluronate Injection for Facial Skin Hydration and Brightness: A Randomized, Controlled, Evaluator‐Blinded Trial
Source: J Cosmet Dermatol. 2026 Jul 1;25(7):e71030. doi: 10.1111/jocd.71030 (PMC13323873; doi:10.1111/jocd.71030)
Supplement: Supplementary file 1 — Table S1: Subject skin assessment scale. Table S2: Improvement grading of transient skin dryness and facial dullness (third‐party evaluator vs. investigator). Table S3: Response rate of improvement in transient skin dryness and facial dullness (third‐party evaluator vs. investigator). [file JOCD-25-e71030-s001.docx]

**Supplementary Materials**

At present, there is no universally accepted standardized scale specifically designed for the comprehensive assessment of transient skin dryness and skin dullness improvement. Therefore, in this clinical trial, the investigators developed an assessment scale based on published literature and clinical practice experience. Considering that skin dryness and skin dullness represent different dimensions of skin quality, they were initially evaluated separately (Table S1). First, the severity of skin dryness and dullness was graded from mild to severe using a 4-point scale (0–3).

During practical application in the study, the scoring results independently assigned by third-party evaluators and investigators for both skin dryness and dullness showed a high degree of consistency (Table S2-3).

Based on this observation, and with reference to the grading principles of the Global Aesthetic Improvement Scale (GAIS), a composite evaluation scale for transient improvement in skin dryness and dullness was established. Compared with baseline, ratings of “very much improved,” “much improved,” and “improved” were defined as effective responses.

**Table S1 Subject Skin Assessment Scale**

| **Assessment of Subject Skin Dryness** | |
| --- | --- |
| 0 | Visually, the skin appears well-hydrated. |
| 1 | Visually, the skin appears slightly dry and tight, with a few fine lines. |
| 2 | Visually, the skin appears moderately dry, with some visible lines. |
| 3 | Visually, the skin appears severely dry, with desquamation and numerous visible lines. |
| **Assessment of Subject Facial Dullness** | |
| 0 | Visually, the skin tone appears even with excellent radiance. |
| 1 | Visually, the skin tone appears relatively radiant, with mild dullness. |
| 2 | Visually, the skin tone shows moderate radiance with noticeable dullness. |
| 3 | Visually, the skin tone appears dull with extremely low radiance. |

**Table S2 Improvement Grading of Transient Skin Dryness and Facial Dullness (Third-Party Evaluator vs. Investigator)**

| **Indicator** | **Treatment Group(Week 12)** | | | **Control Group (Week 12)** | | |
| --- | --- | --- | --- | --- | --- | --- |
|  | Third-Party Evaluator | Investigator | P value | Third-Party Evaluator | Investigator | P value |
| Grade 1: Very marked improvement, n (%) | 5 (2.28) | 5 (2.28) | 0.9710 | 0 (0.00) | 0 (0.00) | 0.6748 |
| Grade 2: Marked improvement, n (%) | 84 (38.36) | 84 (38.36) |  | 2 (0.91) | 2 (0.91) |  |
| Grade 3: Moderate improvement, n (%) | 95 (43.38) | 100 (45.66) |  | 11 (5.02) | 6 (2.74) |  |
| Grade 4: No change, n (%) | 34 (15.53) | 29 (13.24) |  | 198 (90.41) | 203 (92.69) |  |
| Grade 5: Worse than before, n (%) | 1 (0.46) | 1 (0.46) |  | 8 (3.65) | 8 (3.65) |  |
| Total (Missing) | 219 (3) | 219 (3) |  | 219 (9) | 219 (9) |  |

|  | **Treatment Group(Week 12)** | | | **Control Group (Week 12)** | | |
| --- | --- | --- | --- | --- | --- | --- |
| Indicator | Third-Party Evaluator | Investigator | P value | Third-Party Evaluator | Investigator | P value |
| Responders, n (%) | 184 (82.88) | 189 (86.30) | 0.3202 | 13 (5.70) | 8 (3.65) | 0.3061 |
| Non-responders, n (%) | 38 (17.12) | 30 (13.70) |  | 215 (94.30) | 211 (96.35) |  |
| Total (Missing) | 222 (0) | 219 (3) |  | 228 (0) | 219 (9) |  |

**Table S3 Response Rate of Improvement in Transient Skin Dryness and Facial Dullness (Third-Party Evaluator vs. Investigator)**
